# Health anxiety by proxy differs in phenomenology between parents and dog owners

**DOI:** 10.1038/s41598-025-18743-y

**Published:** 2025-09-24

**Authors:** Johanna Lass-Hennemann, Moritz N. Braun, Charina C. Lüder, Tanja Michael, Milena Weigel, Eva M. Zenz, M. Roxanne Sopp

**Affiliations:** 1 Division of Clinical Psychology and Psychotherapy, Department of Psychology, Saarland University, Campus Building A 1.3, Saarbrücken, 66123 Germany; 2 Independent Researcher, Bad Hersfeld, Germany; 3 Independent Researcher, Leipzig, Germany

**Keywords:** Health anxiety by proxy, Hypochondriasis by proxy, Illness-anxiety by proxy, Dog-owner-attachment, Pet parents, Public health, Psychology, Human behaviour

## Abstract

Health anxiety by proxy refers to excessive concerns or preoccupation that a loved one may be suffering from or may acquire a serious illness. Although research with parents suggests that this condition may be highly prevalent, assessment has been limited in terms of attachment figures other than children. Dogs take the role of children in many families and the dog owners and parents show opposing patterns, though the underlying mechanisms are currently unknown attachment between humans and dogs shares important characteristics with the child-parent attachment. Thus, for the first time, we investigated whether health anxiety by proxy is also present in childless dog owners. To this end, we adapted an existing measure of health anxiety by proxy for parents to the situation of dog owners and used the existing parent and adapted dog owner questionnaires to gather data from dog-less parents (*N* = 204) and childless dog owners (*N* = 200). Overall, we found comparable distributions of health anxiety by proxy in both subsamples and high internal consistencies for both questionnaires. Interestingly, dog owners reported higher levels of health anxiety by proxy than parents. In both subsamples, health anxiety by proxy was linked to depressive symptoms and health anxiety, with these associations being stronger in parents than in dog owners. Moreover, while health anxiety by proxy and attachment towards children were negatively associated in parents, we found the opposite association in dog owners. Taken together, these findings provide the first evidence for the existence of health anxiety by proxy in dog owners, while suggesting that the phenomenology of the condition may differ between parents and dog owners.

**Supplementary Information:**

The online version contains supplementary material available at https://doi.org/10.1038/s41598-025-18743-y.

## Introduction

Health anxiety (HA) by proxy has recently been introduced to the published literature to refer to “parents who frequently present their children with various symptoms, despite normal examinations and investigations […] because of a persistent fear that doctors are missing something in their child”^1^. In return, HA by proxy may not only psychologically affect parent and child, visible, for instance, in a positive association between HA by proxy and parental stress due to having a child^2^. Instead, it is likely to expose the child to unnecessary and potentially unpleasant medical examinations and procedures. Moreover, HA by proxy is hypothesized to foster transmission of HA within families^3^, which appears plausible in light of repeated evidence that HA by proxy is positively associated with HA^2,4^. HA by proxy has also been proposed to exist for other attachment figures (e.g. spouses or parents as the proxy;)^2,5^.

However, most research and case reports focus on „children as the proxy“. In contemporary society, modern family concepts have emerged that particularly in cases of childless families, frequently assign dogs a child-like role. Concurrently, the role of the dog owner is analogous to that of a parent. As such, it can be posited that a complementary attachment dynamic typifies both relationships^6^. In these complementary dyads, owners frequently adopt caregiving roles akin to parental figures, with attachment features such as proximity seeking, separation distress, and secure base behavior being observed from both perspectives^7,8^. Given this structural similarity, the psychological investment of dog owners in their dog’s well-being may be analogous to that of parents, especially when the dog shows signs of illness or distress^9^. This provides a foundation for understanding how health-related anxiety, traditionally conceptualized within parent–child dynamics, may also emerge in the context of dog ownership, potentially leading to HA by proxy.

Indeed, clinical experience (both of veterinarians and psychotherapists) has confirmed that HA by proxy may also exist in dog owners and may pose a significant burden on the owner and the pet. Further evidence stems from the to date only published case report describing an owner‘s excessive worries about her dog‘s health^10^. Thus, in the present study, we aimed to investigate for the first time whether HA by proxy is also observable in dog owners by comparing HA by proxy in parents and childless dog owners. To this end, we adapted the Health Anxiety by Proxy Scale (HAPYS)^11,12^, a recently developed measure to assess HA by proxy in parents, to the situation of dog owners. We then employed both the original and the adapted version of the questionnaire to assess HA by proxy in parents and dog owners, respectively. Furthermore, we assessed demographic variables, HA, depressive symptoms, and attachment to one’s child or dog to get a more complete picture of how HA by proxy in parents and dog owners relate. For the parents, we expected HA by proxy to be positively correlated with HA and depressive symptoms (as research has shown that parents of children with chronic diseases report higher levels of depression, for a recent meta-analysis see^13^. Because of the novelty of the construct, a lack of previous studies prevented us from formulating hypotheses regarding the potential correlates of HA by proxy in dog owners.

## Method

### Ethics statement

This study was approved by the Ethics Committee of the Faculty of Empirical Human Sciences and Economics of Saarland University (reference number: 21 − 20) and was performed according to the Declaration of Helsinki.

### Sample

469 individuals participated in the study. Participants were recruited in two different settings: The parent subsample (*N* = 229) was recruited via social media, at local paediatrician or gynaecologist offices, kindergartens, in public parks, and on the street. The dog owner subsample (*N* = 240) was recruited via social media, at local veterinary practices, dog training schools, public parks, and on the street. Prior to data analyses, *N* = 65 participants were excluded because they provided insufficient data or did not meet inclusion criteria. Inclusion criteria for participating parents were that they did not own a dog and that their youngest child was no older than 14 years. For dog owners, the only inclusion criterion was that they did not have a child. All participants were at least 18 years old and indicated that they speak German fluently. The final analysis sample consisted of 404 participants (86.88% female, *M*_age_ = 35.23, *SD*_age_ = 10.15). Among these, two participants of the parent sample failed to complete the attachment questionnaire relating to their child. However, since this data was only considered for exploratory analyses, both participants were retained in the data set for the main analyses. Please note the high proportion of self-identified women in our sample which might limit the generalizability of our findings.

### Questionnaires

#### Health anxiety by proxy: translation and adaption of the health anxiety by proxy scale (HAPYS)

The Health Anxiety by Proxy Scale (HAPYS)^11,12^ is a self-assessment questionnaire for parents that measures HA by proxy related to their own child(ren). The scale consists of 26 items, which are distributed unevenly across the three subscales Thoughts, Feelings, and Behaviour. The items consist of statements that are to be answered on a five-point response scale based on the frequency of occurrence in daily life (ranging from “Not at all” to “A lot” for Thoughts and Feelings and from “Never” to “Most of the time” for Behaviour). The items contain statements on ruminative thoughts (e.g., “I have intrusive unwanted thoughts that my child is seriously ill”), negative feelings (e.g., “I am worried that my child could have a serious illness”) as well as control and avoidance behaviour (e.g., “When I worry about my child’s health: I spend a lot of time seeking information about symptoms and illnesses (online, books, magazines)”) related to HA by proxy. The total score ranges from 26 to 130, with higher scores indicating a higher level of HA by proxy. Items and the response scales were based on existing scales for illness anxiety and illness anxiety by proxy^14,15^. In a first validation study^12^, the HAPYS demonstrated a one factor dimensionality as well as a high internal consistency (α = 0.95) and test-retest reliability after two weeks (*ICC* = 0.91). Convergent validity with parental catastrophising about child pain was good (*r* =.72) and known-groups validity was confirmed.

##### Translation process

Due to a lack of instruments for the assessment of HA by proxy in German, we developed a German version of the HAPYS. To this end, we translated the scale into German in a translation-back-translation process following the guidelines by the International Test Commission (2017). First, two independent translators who were native German speakers with advanced English skills translated the items. These two versions were compared for differences and merged by consensus into one German questionnaire. This version was then back-translated by another independent translator (native German speakers with advanced English skills). The back-translated version was then examined by the first author (JLH) and the last author (MRS) for equivalence. If translations differed from the original scale, they were reviewed again and, if necessary, the German item was adapted to ensure semantic and content equivalence.

##### Adaption to dog owner version

In order to investigate HA by proxy in dog owners, we created a modified version of the HAPYS (the Health Anxiety by Proxy Scale – Dogs, in short: HAPYS-D). For the most part, the adaptation was made by simply changing the word „child“ into the word „dog“. However, some items had to be adapted in terms of content. The following adaptations were made: „Parents“ was replaced with „dog owner“; „doctor“ was replaced with „veterinarian“; „to pass on worries about health“ was replaced with „to transfer health fears“, „I keep asking my child about his/her symptoms“ was replaced with „I keep monitoring my dog regarding his/her symptoms“; „play dates, sports, school trips, dates with friends“ was replaced with „play dates with other dogs, dog sports, trips, walks with other dogs“ and „children’s illnesses“ was replaced with „dog‘s illnesses“. Great care was taken to keep the two HAPYS versions (parents and dog owners) strictly parallel, which was feasible due to the structure of the original questionnaire. The English version of the HAPYS-D is provided in the Supplementary Material. The German version of the HAPYS and HAPYS-D are available from the Authors upon request.

#### Health anxiety

We employed the Whitely Index-14 (WI);^16^ to assess HA in parents and dog owners. The WI is based on the diagnostic criteria of hypochondriasis in DSM IV and consists of 14 items on 3 dimensions: disease phobia (e.g., “Do you often worry about the possibility that you have got a serious illness?”), somatic preoccupation (e.g. “Are you bothered by many aches and pains?”) and disease conviction (e.g.” Is it hard for you to believe the doctor if he tells you that there is nothing to worry about?”). In the WI version that was used in the current study, participants are asked to score items dichotomously (1 = yes or 0 = no), yielding a total score range of 0–14. Reliability and validity of the WI have been tested and confirmed across various studies^17,18^.

#### Depressive symptoms

We employed the Patient Health Questionnaire-9 (PHQ-9; for validation see^19^ to assess depressive symptoms experienced over the past two weeks (e.g. Over the last two weeks how often have you been bothered by the following problems? … 1. Little interest or pleasure in doing things). Responses are scored from 0 = “not at all” to 3 = “nearly every day” with scores ranging from 0 to 27. Symptom severity was assessed according to the sum of all item scores. The internal consistency of the PHQ-9 (α = 0.88 − 0.89) and its retest-reliability are reported to be very high.

#### Attachment to child

We employed the Multiperspective Parent–Child Relationship Questionnaire (M-PCR;)^20^ to assess attachment to one’s child. It contains 14 items on a five-point Likert scale (0 = I disagree; 4 = I agree totally) and provides subscale scores for Affective Bond (e.g. „I believe my child trusts me.”) and Functional-Conflict (e.g. “My child and I are both tense when we do something together”). Higher scores indicate stronger attachment to the child. Both the internal consistency and the retest reliability of the total score have been reported to be very high (see)^20^.

#### Attachment to dog

We employed the German version of the Lexington Attachment to Pets Scale (LAPS; for validation see)^21^ to assess emotional attachment to one’s dog. The scale can be used for cat and dog owners and consists of 23 items (e.g., “My pet understands me” and “My pet and I have a very close relationship.”), which are rated on a 4-point Likert scale. Higher scores indicate a stronger attachment to the pet. Internal consistency of the scale has been reported to be high^22^. We chose the LAPS as it (1) is well-established in the field and (2) measures the strength of the affective bond (as the M-PCR does).

### Procedure

Data collection took place from September 2022 to December 2022 and was conducted online using the platform SosciSurvey^23^. The study was described as a research project on the association between HA related to oneself and one’s dog/child and took around 30 min. Before taking part in the study, participants provided informed consent and were asked to indicate whether they met the inclusion criteria. They then completed demographic questions (age, gender, education level, country of residence, spoken language(s), family status, size of household) followed by the questionnaires assessing health anxiety (WI), depressive symptoms(PHQ-9), and general attachment styles^24^. Results regarding this last questionnaire will be reported elsewhere. Thereafter, participants were asked to complete several questions regarding their child or dog (i.e., age and sex of the child/dog, total number of children/dogs, level of responsibility, whether they are the primary care taker, whether the child/dog lives in the same household, level of experience in taking care of children/dogs). Parents were additionally asked to describe the status of the child (i.e., biological child, adopted child, stepchild) and dog owners were additionally asked to indicate the dog’s breed and the duration of ownership. Eventually, parents and dog owners were asked to complete the HAPYS or the HAPYS-D, respectively, as well as the M-PCR or LAPS, respectively. Parents and dog owners were instructed that – if they had more than one child (under 14 years) or more than one dog –to answer the questionnaire in relation to the child/dog they were most concerned about. If they could not decide, they were asked to randomly select one of their children/one of their dogs. Participants did not receive any compensation in exchange for their participation but were invited to take part in a raffle with gift cards as prizes.

### Data analysis

The data that support the findings of this study are openly available in Mendeley Data at https://data.mendeley.com/datasets/nkn3fwxnrf/1. Data analysis was performed using SPSS 26^25^ and R^26^. The two-sided α level was set to 0.05. Degrees of freedom varied across analyses due to missing data. Parametric tests were used throughout the paper since sample sizes across and within groups were sufficient to assume robustness of these tests against potential violations the normality assumption^27,28^.

In order to investigate differences between parents and dog owners in demographic characteristics and psychopathology, we ran independent-samples *t*-test. In order to optimize testing to detect potential baseline differences between groups, the alpha level was not Bonferroni-corrected for these analyses. HAPYS(-D) scores of parents and dog owners were examined by means of descriptive statistics and by calculating Crombach’s α for both subsamples. To examine differences between the subsamples in the associations between psychopathology and HA by proxy, we ran moderation analyses using the Hayes^29^ PROCESS macro for SPSS. All predictors were mean-centered and 5000 bootstrap resampling was used for the calculation of the confidence intervals. Significant moderation effects were followed up by examining bivariate correlations separately for both subsamples. By contrast, given that we had to use different scales to assess attachment to children and dogs, it was not appropriate to run analyses across subsamples introducing Subsample as a moderator. Therefore, we examined bivariate correlations in each subsample and describe the differences on a descriptive level. In order to account for effects of multiple testing, the alpha level was Bonferroni-corrected for these analyses (*α*_adj_.= 0.0083).

## Results

### Sample characteristics

Sample characteristics of the parent and the dog owner subsamples are reported in Table 1. The dog owners reported a lower mean age and were less likely to be in a relationship at the time of assessment than the parents. These differences were accounted for in the main analyses (see 3.3).

**Table 1:** Demographic characteristics and symptom levels of the parents and the dog owners.

Variables	Parents (*n* = 204)	Dog owners (*n* = 200)	Subsample comparison
	*Count or Mean (SD)*		
**Age**	37.50 (7.29)	32.90 (11.98)	***t*(402) = 4.68**, ***p*** **<.001**
**Sex** Female Male Other	171 32 1	180 18 2	*χ*(2) = 4.45, *p* =.108
**Education years** Less than 9 years 9 10 10 + (advanced professional training) 12/13 (+ university degree)	1 5 20 64 114	4 6 18 51 121	*χ*(4) = 3.64, *p* =.458
**Partner** Yes No	191 13	124 76	***χ*(1) = 58.81**, ***p*** **<.001**
**PHQ-9**	7.42 (5.03)	8.60 (5.68)	***t*(402) = 2.22**, ***p*** **=.027**
**WI**	5.65 (2.28)	5.51 (2.17)	*t*(402) = 0.62, *p* =.536
**HAPYS(-D)**	50.10 (17.52)	57.54 (18.44)	***t*(402) = 4.16**, ***p*** **<.001**
HAPYS(-D)-BH	22.10 (7.41)	24.94 (7.75)	***t*(402) = 3.77**, ***p*** **<.001**
HAPYS(-D)-TH	14.42 (5.70)	17.49 (6.45)	***t*(402) = 5.07**, ***p*** **<.001**
HAPYS(-D)-FE	13.58 (6.11)	15.12 (6.35)	***t*(402) = 2.47**, ***p*** **=.014**

Note. PHQ-9 = Patient Health Questionnaire-9 (Depressive Symptoms). WI = Whitely Index (Health Anxiety). HAPYS = Health Anxiety by Proxy Scale (Health Anxiety by Proxy). HAPYS-D = Health Anxiety by Proxy Scale - Dogs (Health Anxiety by Proxy - Dogs). BH = Behaviour. TH = Thoughts. FE = Feelings.

Neither did the subsamples differ in gender nor regarding the level of education. With respect to psychopathology, the dog owners reported higher depression and HA by proxy scores than the parents, whereas the subsamples did not differ in HA. 18.5% of the dog owners and 22.1% of the parents screened positive for HA (WI score ≥ 8;)^16^, while 34.5% of the dog owners and 28.9% of the parents screened positive for depressive symptoms (PHQ-9 score ≥ 10;)^24^.

### Examination of the HAPYS(-D) scores of the parents and the dog owners

**Fig. 1:**
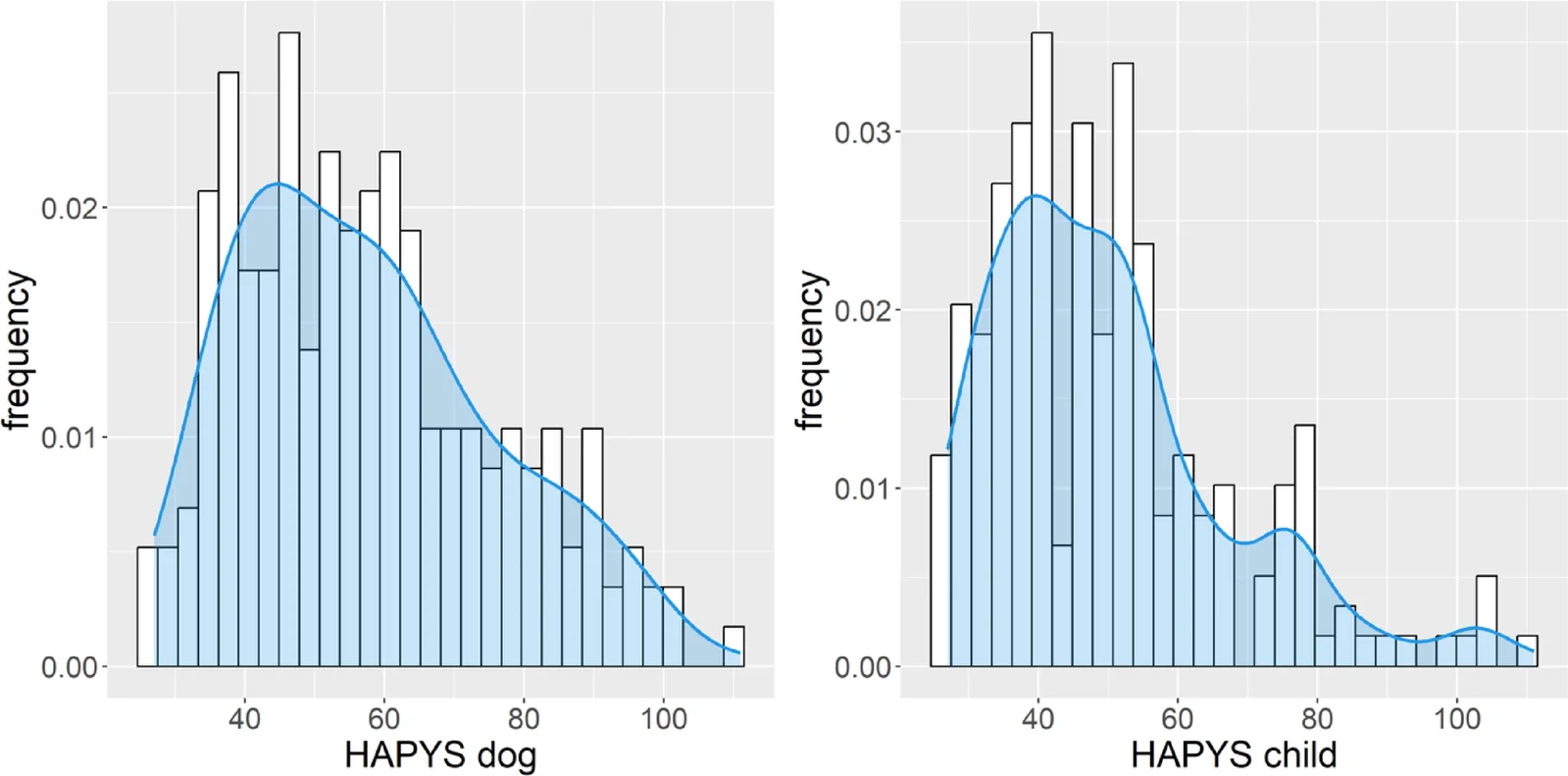
Distributions of the HAPYS and HAPYS-D scores of the parents and the dog owners, respectively. *Note.* Parents: Skewness = 1.14, Kurtosis = 1.18. Dog owners: Skewness = 0.57, Kurtosis = −0.41.

The distribution of the HAPYS(-D) scores is depicted in Fig. 1. As to be expected, distributions were right-skewed in both subsamples, reflecting the fact that participants were recruited in non-clinical settings. The internal consistency of the scale was high across both subsamples (parents: α = 0.951, dog owners: α = 0.938).

### Associations between health anxiety by proxy and other psychopathological symptoms

#### Health anxiety

In order to examine the association between HA and HA by proxy across subsamples, we ran moderation analyses controlling for age and partnership status. The model including HA (i.e., WI scores) as the predictor, age and partnership status as covariates, Subsample as the moderator, and HA by proxy (i.e., HAPYS(-D) scores) as the dependent variable was found to be significant (*F*(5,398) = 19.99, *p* <.001, R² = 0.201). In line with our hypotheses, greater HA predicted higher HA by proxy (*B* = 2.87, *t* = 7.73, *p* <.001, CI 95% [2.14, 3.60]). Moreover, in line with our previous analyses, dog owners reported higher HA by proxy than parents (*B* = −7.79, *t* = 4.27, *p* <.001, CI95% [−11.38, −4.20]). Moreover, there was a significant moderation effect, indicating that the association between HA and HA by proxy differed significantly between the subsamples (*B* = 2.55, *t* = 3.44, *p* <.001, CI 95% [1.09, 4.01]). This effect accounted for an additional 2.4% of the variance. In order to further characterize this moderation effect, we examined correlations between HA and HA by proxy separately for the two subsamples. The association was stronger for the parents (*r*(204) = 0.540, *p* <.001) than for the dog owners (*r*(200) = 0.178, *p* =.012). Neither age (*B* = −0.16, *t* = 1.91, *p* =.057, CI 95% [−0.32, 0.005]) nor partnership status (*B* = 2.19, *t* = 2.15, *p* =.309, CI 95% [−2.04, 6.42]) were significantly associated with HA by proxy.

##### Depressive symptoms

In order to examine the association between depressive symptoms and HA by proxy across subsamples, we ran moderation analyses controlling for age and partnership status. The model including depressive symptoms (i.e., PHQ-9 scores) as the predictor, age and partnership status as Ccvariates, Subsample as the moderator, and HA by proxy (i.e., HAPYS(-D) scores) as the dependent variable was found to be significant (*F*(5,398) = 21.30, *p* <.001, R² = 0.211). In line with our hypotheses, more depressive symptoms predicted higher HA by proxy (*B* = 1.38, *t* = 8.87, *p* <.001, CI95% [1.08, 1.69]). Again, it was confirmed that dog owners reported higher HA by proxy than parents (*B* = −6.32, *t* = 3.49, *p* <.001, CI95% [−9.89, −2.76]). Moreover, there was a significant moderation effect, indicating that the association between depressive symptoms and HA by proxy differed significantly between the subsamples (*B* = 0.73, *t* = 2.37, *p* =.019, CI95% [0.12, 1.33]). This effect accounted for an additional 1.2% of the variance. In order to further characterize this moderation effect, we examined correlations between depressive symptoms and HA by proxy separately for the two subsamples. The association was stronger for the parents (*r*(204) = 0.503, *p* <.001) than for the dog owners (*r*(200) = 0.308, *p* <.001). However, differences were less marked than for the association between HA by proxy and HA. Neither age (*B* = −0.10, *t* = 1.07, *p* =.287, CI95% [−0.25, 0.08]) nor partnership status (*B* = 2.91, *t* = 1.35, *p* =.178, CI95% [−1.33, 7,14]) were significantly associated with HA by proxy.

### Exploratory analyses: association between health anxiety by proxy, psychopathology, and attachment to the proxy

In order to examine the association between HA by proxy, psychopathology, and attachment to the respective proxy (i.e., child or dog), we examined bivariate correlations between these variables separately for each subsample. For the parents, HA by proxy was negatively correlated with attachment to one’s child (*r*(202) = − 0.452, *p* <.001), reflecting that the attachment to one’s child might be a protective factor against the development of HA by proxy. In a similar vein, lower HA (*r*(202) = − 0.264, *p* <.001) and lower depressive symptoms (*r*(202) = − 0.451, *p* <.001) were linked to stronger attachment to one’s child. By contrast, for the dog owners, we found a positive link between HA by proxy and attachment to one’s dog (*r*(200) = 0.397, *p* <.001). Similarly, higher depressive symptoms were linked to stronger attachment to one’s dog (*r*(200) = 0.226, *p* <.001). However, HA and attachment to one’s dog were not significantly correlated (*r*(200) = 0.021, *p* =.767).

## Discussion

HA by proxy is a newly described phenomenon where parents worry excessively that their child suffers from a serious disease^5^. So far, research on HA by proxy has exclusively focused on children as the proxy^2,5^. In the present study, we aimed to investigate for the first time whether HA by proxy is also observable in dog owners and, if so, how HA by proxy in dog owners relates to HA by proxy in parents by comparing HA by proxy and its correlates in parents and childless dog owners.

Overall, we found comparable distributions of HA by proxy in both subsamples. Moreover, both the HAPYS and the HAPYS-D showed high internal consistencies. This indicates that HA by proxy is also present in childless dog owners. Interestingly, overall, dog owners reported higher levels of HA by proxy than parents. One potential explanation for this phenomenon is that, although humans form strong bonds with their dogs^30^, the human ability to read dogs’ and other pets’ communicative signals is far from perfect^31–34^.This could lead to insecure and ruminative thoughts about their dog’s health, which may result in increased control behaviors and higher HA by proxy scores in dog owners. Another potential explanation is that at least some of the dog owners might have gotten their dog to promote their mental health and wellbeing^35^. This in turn might mean that they are more prone to mental health problems in the first place and thus, the development of HA by proxy. This explanation receives support from our finding that the dog owners reported significantly more depressive symptoms than the parents.

In both subsamples, a higher level of HA by proxy was associated with a higher level of HA and more depressive symptoms. Although both associations were significantly stronger for the parents than the dog owners, both subsamples exhibited the same pattern. In light of the comparatively weaker association between HA by proxy and psychopathology in dog owners, one might argue that HA by proxy in dog owners has less clinical relevance. On the other hand, however, dog owners reported higher levels of HA by proxy, which suggests that HA by proxy in dog owners may warrant clinical attention. Future studies assessing HA by proxy in dog owners and parents should also include broader measures of psychopathology and health to shed light on this issue.

In addition to its possible impact on the psychological well-being of dog owners, HA by proxy is also relevant from a veterinary perspective. Studies on HA by proxy in parents have shown that parents with high levels of HA by proxy may seek excessive pediatric help and insist on unnecessary medical examinations^2–4^. Furthermore, they tend to frequently change their child’s physicians due to mistrust of their diagnoses. A published case report^10^ and clinical experience show that dog owners with high levels of HA by proxy exhibit similar behaviours. Future research should focus on the veterinary perspective of HA by proxy in pet owners to better understand the challenges veterinarians face with owners exhibiting this behaviour.

Interestingly, dog owners and parents showed opposite patterns with regard to the association between HA by proxy and attachment to one’s child/dog. While this association was negative for the parents (i.e., stronger attachment to one’s child was associated with less HA by proxy), the association was positive for the dog owners (i.e., stronger attachment to one’s dog was associated with higher HA by proxy). This suggests that the phenomenology of HA by proxy may differ between parents and dog owners, which can be considered and explained from different perspectives.

On the one hand, a fundamental difference may lie in the underlying goals associated with parenting versus dog ownership. In the context of parenting, caregiving goals are dynamic and adapt over time^36^: While early parenthood is focused on protection and provision, a central long-term objective is to foster the child’s independence and autonomy. In contrast, dog ownership is generally oriented around continuous care. Through domestication, dogs are removed from their natural environments and, consequently, from the opportunity to develop or maintain autonomous survival skills. As such, the caregiving relationship remains one of sustained dependence. Strong attachment in both roles may be associated with a desire to do one’s best as a caregiver^37^ in order to achieve the goals described. Parents with a strong attachment bond may strive to promote their child’s independence and a sense of security. As such, they may experience lower levels of HA by proxy, as fostering autonomy requires a certain degree of emotional distance and confidence in the child’s resilience. In contrast, dog owners with a strong attachment bond may exhibit an intensified desire to provide continuous care, including ongoing protection and monitoring. The well-intentioned goal of ensuring continued care in dog owners may contribute to higher levels of HA by proxy. Thus, while both parents and dog owners may be motivated by a similar internal standard of being a “good caregiver,” the divergent goals of caregiving may possibly explain the contrasting results.

On the other hand, the former result (i.e., a negative association between attachment to one’s child and HA by proxy) could be interpreted in light of the recent finding that parents with distressing worries about their child’s health report feelings of mistrust derived from their HA by proxy that invades their relationships with their children^12^. Arguably, however, our findings could also be interpreted as suggesting that attachment to one’s own child is a protective factor against the development of HA by proxy in parents. An interpretation that could be generalised to attachment to one’s own child as a protective factor against the development of psychopathology more broadly, given our findings that stronger attachment to one’s own child was also associated with fewer depressive symptoms and lower HA. However, it could also be the case that less psychopathology in parents fosters the attachment to one’s child. Which (if any) of these interpretations holds true, however, cannot be solved with our cross-sectional design. Future research should test these interpretations using longitudinal designs.

By contrast, the positive association between attachment to one’s dog and HA by proxy and our finding that stronger attachment to one’s dog was linked to higher depressive symptoms are in line with previous studies linking a stronger attachment to one’s dog to higher levels of psychopathology^38,39^. This – at first glance – confusing association might be explained by the relationship between attachment to pets and attachment to humans. Previous research has given some hints that a strong attachment to one’s pet might reflect a compensatory attachment strategy for humans who were not able to establish secure relationships to other humans. This idea is inspired by the previous finding that insecure attachment to humans fully mediates the repeatedly observed negative association between emotional attachment to pets and mental health burden^39^.

Importantly, although unselected, both of our subsamples mainly consisted of self-identified women. Thus, it remains an open question whether our findings and their presentation also hold for gender-balanced samples and samples that mainly consist of self-identified men or are specific to female parents and female childless dog owners. We call future research to scrutinize this.

### Limitations

The study has several limitations worth noting. First, the cross-sectional design does not allow us to infer a causal relationship between study variables. This is especially true for the interpretation of attachment to one’s child as a protective factor. To directly test this idea, longitudinal designs could be employed in future studies. Second, due to the absence of a suitable measure that allows the assessment of both attachment to a child and a dog within a single questionnaire, we employed two different measures, one to assess attachment to a dog (the LAPS) and one to assess attachment to a child (the M-PCR). Importantly, both the LAPS and the M-PCR are designed to measure the strength of the affective bond (between dog owner and dog and parent and child, respectively). However, the M-PCR also contains a Functional Conflict subscale. While the former parallel supports the comparability of the two questionnaires, the latter difference might limit the comparability of the findings from our exploratory analyses regarding attachment in parents and dog owners. Future research should further scrutinize our findings by studying their robustness to the employment of different attachment measures. Third, we decided to solely include childless dog owners and dog-less parents in our study. Thus, we excluded individuals that are both parents and dog owners. We did so to be able to investigate the phenomenology of HA by proxy with children and dogs as attachment figures without one confounding the other. It is unclear if and how this decision influenced the outcomes of our study and if the differences in phenomenology of HA by proxy that we found between our two subsamples hold within a single sample of dog-owning parents. To clarify this, future studies could explore HA by proxy with children and dogs as attachment figures in a sample of individuals that are both parents and dog-owners and compare the respective findings with the findings from childless dog owners and dog-less parents. Likely, the role of dogs differs in families with children and, thus, the phenomenology of HA by proxy may also vary in this context. Fourth, concerns about the data quality of online studies have been raised^40^. However, it is important to note that while assessment was conducted online, participants were mostly recruited offline. Moreover, participation was voluntary and uncompensated, which likely attracted participants with a genuine interest in the study. Fifth, we employed self-report measures to assess symptoms, which may be influenced by memory biases. Sixth, while our sample was unselected, it consisted mainly of self-identified women. Thus, our findings might reflect the attitudes and experiences of women who were interested in the topic of our study. Moreover, subsamples differed in age and partnership status. While we did our best to control for these potential confounders in our analyses, we cannot fully exclude the existence of confounding effects. To allow for generalization of the findings to the general population, future studies should replicate our findings in more diverse samples as well as in selected clinical samples.

## Conclusion

To the best of our knowledge, our study is the first to find evidence for HA by proxy in a population other than parents. Our data suggest that while HA by proxy exists in both parents and dog owners, the phenomenology of the condition may differ between parents and dog owners: Dog owners report overall higher levels of HA by proxy, while HA by proxy is more strongly associated with other psychopathological symptoms in parents. Pets – especially dogs – take the role of children in many families and our findings, together with earlier research, suggest that we might need to pay more attention to the anxiety symptoms that dog owners may experience and to the potential harm that these may pose on their dogs. Future studies are needed to get a more comprehensive picture of HA by proxy in dog owners and should also focus on HA by proxy with attachment figures other than children or dogs (e.g., parents, partners, cats, and horses).

## Supplementary Information

Below is the link to the electronic supplementary material.

## Data Availability

The data that support the findings of this study are openly available in Mendeley Data at https://data.mendeley.com/datasets/nkn3fwxnrf/1.
