# Correction: Health anxiety by proxy differs in phenomenology between parents and dog owners

**DOI:** 10.1038/s41598-026-67592-w

**Published:** 2026-09-02

**Authors:** Johanna Lass-Hennemann, Moritz N. Braun, Charina C. Lüder, Tanja Michael, Milena Weigel, Eva M. Zenz, M. Roxanne Sopp

**Affiliations:** 1 Division of Clinical Psychology and Psychotherapy, Department of Psychology, Saarland University, Campus Building A 1.3, Saarbrücken, 66123 Germany; 2 Independent Researcher, Bad Hersfeld, Germany; 3 Independent Researcher, Leipzig, Germany

Correction to: *Scientific Reports* https://doi.org/10.1038/s41598-025-18743-y, published online 24 September 2025

The original version of this Article contained an error where Milena Weigel and Eva M. Zenz were omitted from the author list. As a result, the author list and affiliations now read:

Johanna Lass-Hennemann^1^, Moritz N. Braun^1^, Charina C. Lüder^1^, Tanja Michael^1,4^, Milena Weigel^2^, Eva M. Zenz^3^ & M. Roxanne Sopp^1,4^

^1^Division of Clinical Psychology and Psychotherapy, Department of Psychology, Saarland University, Campus Building A 1.3, Saarbrücken 66123, Germany. ^2^Independent Researcher, Bad Hersfeld, Germany. ^3^Independent Researcher, Leipzig, Germany. ^4^These authors jointly supervised this work: Tanja Michael and M. Roxanne Sopp.

Furthermore, the Author contributions section now reads:

J.L.: Conceptualization, Project Administration, Methodology, Writing (Original Draft, Review and Editing); M.N.B.: Methodology, Investigation, Writing (Original Draft, Review and Editing); C.C.L.: Methodology, Investigation, Writing (Review and Editing), Conceptual Input; T.M.: Supervision, Resources, Writing (Review and Editing); M.W.: Methodology, Investigation; E.M.Z.: Methodology, Investigation; M. R.S.: Supervision, Formal Analysis, Visualization, Writing (Original Draft, Review and Editing).

Finally, the Supplementary Material 2 file has been updated with the added author names.

The original Article and Supplementary Information files have been corrected.

## Supplementary Information

Below is the link to the electronic supplementary material.

