# Supplementary material for: Health anxiety by proxy differs in phenomenology between parents and dog owners
Source: Sci Rep. 2025 Sep 24;15:32692. doi: 10.1038/s41598-025-18743-y (PMC12460664; doi:10.1038/s41598-025-18743-y)
Supplement: Supplementary file 2 — Supplementary Material 2 [file 41598_2025_18743_MOESM2_ESM.docx]

**Health anxiety by proxy differs in phenomenology between parents and dog owners**

*Johanna Lass-Hennemann*^1^, Moritz N. Braun^1^, Charina C. Lüder**^1^, Tanja Michael*°*^1^ and M. Roxanne Sopp*°*^1^*

*^1^Division of Clinical Psychology and Psychotherapy, Department of Psychology, Campus Building A 1.3 Saarland University, 66123 Saarbrücken, Germany*

° Shared last authorship

***** Correspondence:

Dr. Johanna Lass-Hennemann

Email: lass-hennemann@mx.uni-saarland.de

****** Correspondence on behalf of Dr. Johanna Lass-Hennemann:

Charina C. Lüder

Email: charina.lueder@uni-saarland.de

**SM1 HAPYS-D (English version)**

HAPYS-D (English version)

The following statements are about thoughts, feelings and behaviour *dog owners* may experience when they are worried about their *dog*’s health. If you have more than one *dog*, we ask you to answer the questionnaire based on the *dog* whose health you may have the most worries about or choose a *dog* at random.

Please note, there are no wrong or right answers, therefore answer each item in terms of how frequently the statement applies to you.

Do not use too much time on the questionnaire, but answer what first come to mind.

Are you the *owner* of the *dog*?______________

*Dog*’s age _________________________________________

All in all, how much are you worried about your *dog*’s health?

***Thoughts*** (Scale: Not at all, A little, Some, Quite a lot, A lot)

1. I keep having thoughts about my *dog*’s health

2. It is difficult to put thoughts about my *dog*’s health out of my mind

3. I have thoughts that symptoms like pain, fatigue or discomfort etc. in my *dog* are signs of

serious illness

4. People close to me think I worry too much about my *dog*’s health

5. I think I worry more about my *dog*’s health compared to other *dog owners*

6. I have intrusive unwanted thoughts that my *dog* is seriously ill

7. I have thoughts that the *veterinarian* might be wrong if he/she tells me there is nothing to worry about

***Feelings*** (Scale: Not at all, A little, Some, Quite a lot, A lot)

8. I am worried that my *dog* could have a serious illness

9. I am worried about passing worries about health on to my *dog*

10. I am worried that I am missing a serious illness in my *dog*

11. I am worried that the *veterinarian* is missing a serious illness in my *dog*

12. If I hear or read about illness, I get worried that my *dog* may suffer from the same illness

13. Worrying about my *dog*’s health makes me feel sad

14. I feel guilt or shame because of worries about my *dog*’s health

15. I feel like I am losing control if I worry about my *dog*’s health

***Behaviour*** (Scale: Never, Rarely, Some-times, Often, Most of the time)

When I worry about my *dog*’s health:

16. I spend a lot of time seeking information about symptoms and illnesses (online, books, magazines)

17. I repeatedly seek reassurance from my partner or close family members

18. I repeatedly seek reassurance from my *veterinarian* or other health professionals

19. I only feel briefly reassured, if I seek help from health professionals

20. I keep checking my *dog*’s body for signs of illness and/or *keep* *monitoring* my *dog* *regarding* his/

her symptoms

21. I feel a need to check my *dog*’s body for signs of illness

22. I am more inclined than other dog owners to limit my *dog* in various activities (play dates *with other dogs*, *dog* sports, trips, *meetings with other dogs*)

23. I pay more attention to his/her behaviour

24. I avoid reading or hearing about *dog*’s illnesses

25. I restrict my *dog*’s exposure to sick *dogs*.

26. I try to distract myself to get rid of the worries (e.g. think about something else, listen to music or

watch TV)

--------------

***Impact***

Overall, do you think that your worries about your *dog*’s health are a problem? No; Yes, a little bit; Yes, quite a bit; Yes, a great deal

If yes, how long have the worries been present? (circle) 0–6 months; 6–12 months; Over a year

If yes, do you think the worries are a burden for:

You? No; Yes, a little bit; Yes, quite a bit; Yes, a great deal

Your *dog*? No; Yes, a little bit; Yes, quite a bit; Yes, a great deal

Your family as a whole? No; Yes, a little bit; Yes, quite a bit; Yes, a great deal

If yes, do the worries affect your daily life in relation to:

Your partner or close family? No; Yes, a little bit; Yes, quite a bit; Yes, a great deal

Work or study? No; Yes, a little bit; Yes, quite a bit; Yes, a great deal

Social relations? No; Yes, a little bit; Yes, quite a bit; Yes, a great deal

Do you know how your worries about your *dog*’s health started?

Yes; No

If yes, describe it here:

**SM2 Participants' Declaration of Consent and Ethical Considerations**

Dear Participants,

To ensure the quality and reliability of the data collected, we ask you to answer the preliminary questions carefully and honestly. We also encourage you to read the participant information sheet carefully beforehand.

In this study, we investigate whether health anxiety by proxy is also present in childless dog owners. For this purpose, we adapted an existing measure of health anxiety by proxy for parents to the context of dog owners. The adapted questions will be presented to you throughout the survey - please answer all questions truthfully. The entire process will take approximately 30 minutes.

Your participation is entirely voluntary. You may withdraw from the study at any time without providing a reason and without experiencing any disadvantages.

Participation involves minimal risk, mainly potential emotional discomfort when answering questions about your well-being. However, your participation may help enhance understanding of health anxiety by proxy.

All your responses will be treated confidentially and used exclusively for research purposes. The scientific documentation, storage, analysis, and potential sharing of the data with other research groups will only occur in a pseudonymized form (i.e., without any information that could allow identification of your person).

The study has been reviewed and approved by the Ethics Committee of Saarland University (reference number: 21-20). All procedures comply with the ethical standards outlined in the Declaration of Helsinki.

**Declaration of Consent**

I hereby agree to voluntarily participate in the following study. I confirm that I have carefully read and understood the provided information, including the participant information sheet and the declaration on the handling of collected data. I am aware of the study’s aim, procedure, and conduct. I was given sufficient time to reconsider my participation and understand that I can ask further questions at any time. I acknowledge that I can withdraw from the study at any time or choose not to answer any question without providing a reason and without experiencing any disadvantages.

If you have any questions or need further clarification or if you experience any distress during or after the study and need support, please contact us at [lass-hennemann@mx.uni-saarland.de](mailto:lass-hennemann@mx.uni-saarland.de)

Thank you for your participation!
